# Complement blockade with eculizumab to treat acute symptomatic humoral rejection after heart transplantation

**DOI:** 10.1111/xen.12726

**Published:** 2022-01-10

**Authors:** Patrick Yerly, Samuel Rotman, Julien Regamey, Vincent Aubert, Stefania Aur, Matthias Kirsch, Roger Hullin, Manuel Pascual

**Affiliations:** ^1^ Service of Cardiology Lausanne University Hospital (CHUV) and Lausanne University Lausanne Switzerland; ^2^ Service of Clinical Pathology Lausanne University Hospital (CHUV) and Lausanne University Lausanne Switzerland; ^3^ Service of Immunology and Allergology Lausanne University Hospital (CHUV) and Lausanne University Lausanne Switzerland; ^4^ Service of Cardiac Surgery Lausanne University Hospital (CHUV) and Lausanne University Lausanne Switzerland; ^5^ Center for Organ Transplantation Lausanne University Hospital (CHUV) and Lausanne University Lausanne Switzerland

**Keywords:** antibody‐mediated rejection, complement, eculizumab, heart transplantation, xenotransplantation

## Abstract

Antibody‐mediated rejection (AMR) is a major barrier preventing successful discordant organ xenotransplantation, but it also occurs in allotransplantation due to anti‐HLA antibodies. Symptomatic acute AMR is rare after heart allograft but carries a high risk of mortality, especially >1 year after transplant. As complement activation may play a major role in mediating tissue injury in acute AMR, drugs blocking the terminal complement cascade like eculizumab may be useful, particularly since “standards of care” like plasmapheresis are not based on strong evidence. Eculizumab was successfully used to treat early acute kidney AMR, a typical condition of “active AMR,” but showed mitigated results in late AMR, where “chronic active” lesions are more prevalent. Here, we report the case of a heart recipient who presented with acute heart failure due to late acute AMR with eight de novo donor‐specific anti‐HLA antibodies (DSA), and who fully recovered allograft function and completely cleared DSA following plasmapheresis‐free upfront eculizumab administration in addition to thymoglobulin, intravenous immunoglobulins (IVIG), and rituximab. Several clinical (acute onset, abrupt and severe loss of graft function), biological (sudden high‐level production of DSA), and pathological features (microvascular injury, C4d deposits) of this cardiac recipient are shared with early kidney AMR and may indicate a strong role of complement in the pathogenesis of acute graft injury that may respond to drugs like eculizumab. Terminal complement blockade should be further explored to treat acute AMR in recipients of heart allografts and possibly also in recipients of discordant xenografts in the future.

## 1 INTRODUCTION

Antibody‐mediated rejection (AMR) is one of the major barriers preventing discordant organ xenotransplantation because of naturally occurring antibodies against xenoantigens (like anti‐pig‐Gal in humans and nonhuman primates), but it also occurs in allotransplantation mainly due to anti‐HLA antibodies.^1^ Symptomatic acute AMR is quite rare after heart allograft but it remains a major therapeutic challenge, with unpredictable response to treatment and high risk of mortality.^2^, ^3^, ^4^, ^5^ Currently available therapeutic options are numerous but they are mostly based on retrospective and uncontrolled studies collected from small heterogeneous patient populations (e.g., acute and chronic AMR, early and late AMR, and so on) with inconsistent and conflicting results.^2^ The disparity observed in the treatment effects may, however, also arise from the variability of the possible interactions between donor‐specific antibodies (DSA) and allograft endothelial cells (e.g., complement cascade activation, direct signaling via HLA molecules, Fc gamma receptor‐dependent cellular effects), which may not be equally relevant in all AMR cases, and which are differently targeted by each particular therapy.

One of the most important pathogenic processes leading to tissue injury in allograft and xenograft acute AMR is the activation of the classical pathway of the complement cascade by endothelial‐bound DSA, with ensuing C4d deposition, microvascular inflammation, thrombosis, and capillary obstruction.^6^, ^7^ In that setting, the use of drugs inhibiting complement‐like eculizumab is appealing. Eculizumab is a humanized monoclonal antibody that binds to the C5 complement component, preventing its conversion to anaphylatoxin (C5a) and blocking the constitution of the cell membrane attack complex (C5b‐9), which finally results in a powerful inhibition of the complement cascade.

In kidney allotransplantation, eculizumab has been successfully used to prevent acute AMR in sensitized high‐risk recipients^8^ or to treat early acute AMR together with plasmapheresis in sensitized recipients with abrupt posttransplant DSA rise and rapidly evolving allograft dysfunction,^9^ a condition known to be complement‐dependent. Of note, our group also reported successful eculizumab administration instead of extracorporeal antibody removal,^10^, ^11^ with the idea that immediate direct neutralization of the pathogenetic effects of DSA by terminal complement blockade may favorably replace the more invasive, cumbersome, and slowly working plasmapheresis or immunoadsorption procedures.^6^ Of importance also, therapeutic attempts with eculizumab were not all successful, for example, with mitigated results in late AMR^12^ or when eculizumab was used as a rescue therapy after other treatments failed.^13^

In heart transplantation (HTx), eculizumab given during 3 months after transplant was very recently shown to better prevent AMR than peri‐operative plasmapheresis and intravenous immunoglobulins (IVIG) in highly sensitized recipients with positive virtual crossmatch.^14^ However, the experience with eculizumab for established AMR is still preliminary and mainly restricted to refractory AMR after plasmapheresis.^15^ To our best knowledge, the only report about eculizumab use is a retrospective analysis of 14 pediatric heart recipients.^16^ Only 11 of them had endomyocardial biopsy (EMB) proven AMR, the remaining three receiving eculizumab for AMR prevention after HTx with a positive crossmatch. The global outcome was poor, with 50% early mortality.

Here, we report the case of a heart allotransplant recipient with sudden acute heart failure due to late acute AMR with eight de novo quickly rising DSA who fully recovered allograft function following plasmapheresis‐free eculizumab administration, and who completely cleared circulating DSA with thymoglobulin, IVIG, and rituximab subsequently given to suppress DSA production. We suggest that blocking the terminal complement pathway may become a useful strategy to treat cardiac acute AMR associated with abruptly occurring severe allograft dysfunction, intense DSA production, evident microvascular injury, and C4d deposition on EMB. In the future, similar therapeutic strategies may also apply to treat AMR in discordant xenotransplantation.

## 2 CASE REPORT

**FIGURE 1:**
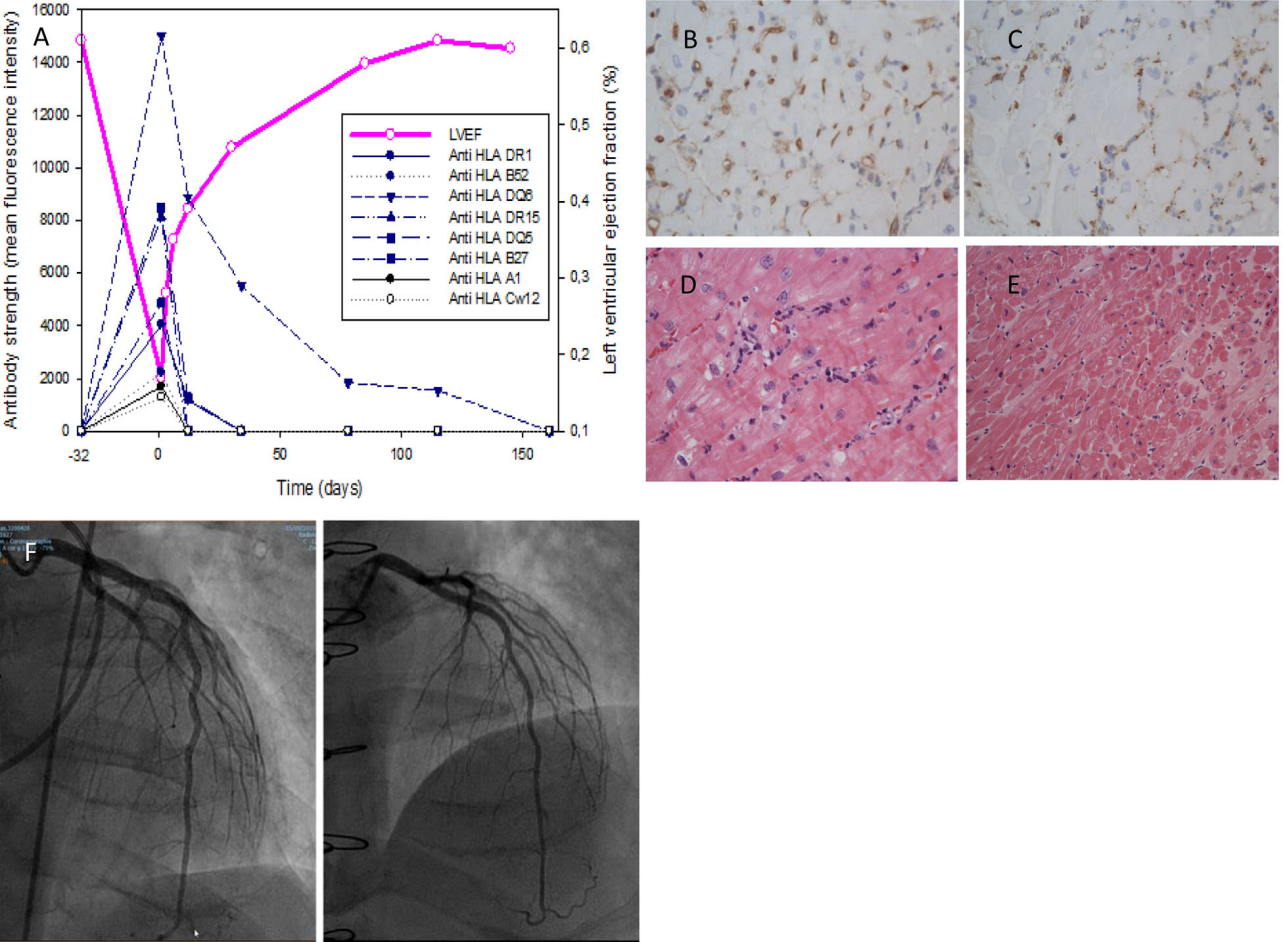
(A) Allograft function (LVEF) and donor‐specific antibodies (DSA) strength (MFI) over time. Day 1 = AMR diagnosis. Day ‐32 = time of routine evaluation 1 year after transplant. (B) C4d staining (immunochemistry) showing intense complement deposition in capillaries = immune‐pathological criterium for AMR (pAMR I+). (C) CD68 staining (immunochemistry) showing intra‐capillary macrophage deposition = immune‐pathological criterium for AMR (pAMR I+). (D) Hematoxillin‐eosin staining showing intra‐vascular activated mononuclear cells = morphologic criterium for AMR (pAMR H+) (pAMR I+ and pAMR H+ together = pAMR2). H&E, 400x. (E) Hematoxillin‐eosin staining. Control biopsy (5 months) showing complete AMR recovery. H&E, 200x (F) Coronary angiography 32 days before AMR with no CAV (G) Coronary angiography 5 months after AMR with no CAV

The patient is a 43‐year‐old male who presented with severe heart failure due to de novo dilated cardiomyopathy diagnosed 5 months before HTx. Because of severely depressed left ventricular ejection fraction (LVEF) at presentation (9% on cardiac MRI), severe functional mitral regurgitation, and poor response to medical therapy, the patient was rapidly listed for HTx and a left ventricular assist device (LVAD) (Heartmate 3, Abbott cardiovascular, Chicago, IL, USA) was inserted 4 weeks after admission as a bridge to transplantation. Screening for HLA antibodies class I and II (single antigen bead assay by Luminex technology) was negative (<1000 MFI) before LVAD and slightly positive 3 weeks after for one epitope later classified as non‐donor‐specific (anti‐Cw15 1500 MFI). HTx occurred 16 weeks after LVAD placement. No detectable anti‐HLA antibodies were present at the time of transplant. Induction immunosuppression included basiliximab 20 mg at days 0 and 4 and five daily doses of intravenous methylprednisolone (500 mg at day 0 to 125 mg at day 4). Maintenance immunosuppression consisted of ciclosporin, mycophenolate, and oral prednisolone as per our standard protocol. At 1 year, the patient was doing well (NYHA2) and underwent per‐protocol investigations showing 61% LVEF on echocardiography, no circulating DSA, no rejection (0R/pAMR0) on EMB, and no cardiac allograft vasculopathy (CAV) on coronary angiography (Figure 1). Thirty‐two days later, during per‐protocol prednisolone tapering from 10 to 5 mg, he complained of progressing dyspnea over 48 h and was admitted with severe allograft dysfunction (LVEF 17%) and suspected acute rejection. Pending EMB scheduled for the next morning, the patient received 5oo mg methylprednisolone and thymoglobulin 1 mg/kg but still deteriorated rapidly, with cardiogenic shock needing high‐dose inotropic support 12 h after initiating therapy (cardiac index 1.9 L/min/m^2^ and SvO_2_ 49% on dobutamine 400 μg/min). The EMB revealed a pAMR2 rejection with four de novo circulating anti‐HLA class‐I DSA (anti‐B27/B52/A1/Cw12 at 4041/2234/1685/1298 MFI) and four de novo anti‐HLA class‐II DSA (anti‐DQ6/DQ5/DR15/DR1 at 15011/8637/8477/4866 MFI) (Figure 1). The patient was immediately treated with eculizumab 900 mg (days 1 and 8) with complete complement inhibition during 17 days (CH‐50 < 10%). Methylprednisolone 500 mg and thymoglobulin 1 mg/kg were continued for 2 additional days, IVIG 2 g/kg was started on day 4 and repeated six times monthly thereafter, and rituximab 375 mg/m^2^ was finally introduced on day 11 and repeated on day 22.

Hemodynamics improved strikingly quickly after eculizumab initiation with cardiac index and SvO_2_ measured at 2.9 L/min/m^2^ and 58% on reduced dobutamine dosing (300 μg/min) 18 h after infusion, and at 3.2 L/min/m^2^ respectively 64% on dobutamine 200 μg/min, 36 h after infusion. Inotropic support terminated on day 5 and LVEF increased to 28%, 35%, 39%, and 47% on days 3, 6, 12, and 30, respectively, before normalization on day 85 (58%). DSA progressively decreased over 4 weeks and was undetectable for 6 months after treatment with no CAV at angiography (Figure 1).

Importantly, the patient also received prophylactic phenoxymethylpenicillin to prevent *Neisseria meningitidis* infection between the first dose of eculizumab and CH‐50 recovery as well as valganciclovir and trimethoprim‐sulfamethoxazole to prevent *Cytomegalovirus* and *Toxoplasma gondii* reactivation during 3 months. No significant infectious or oncologic complication occurred after rejection therapy.

## 3 DISCUSSION

In this case of acute pathologically proven AMR with severe allograft dysfunction, a poly‐therapy consisting of steroids, thymoglobulin, IVIG, and upfront eculizumab was successfully used to block tissue injury and suppress DSA production. Although we acknowledge that all agents might have contributed to the successful outcome, the short delay observed between eculizumab infusion and hemodynamic improvement in a patient actually deteriorating after methylprednisolone and thymoglobulin administration suggests that eculizumab played a central role in AMR resolution. Furthermore, as far as plasmapheresis or immunoadsorption was not used in this patient and as far as IVIG was only started on day 4, complement blockade with eculizumab was the sole undertaken intervention that directly targeted acute AMR before early improvement.

This finding underscores the key role complement can play in mediating tissue injury in AMR, both in allo‐ or xenotransplantation. Indeed, the currently contemplated pig‐to‐human kidney transplantation trials^1^ would not be possible without introducing at least one human complement‐regulatory transgene into the engineered pigs to provide some protection against AMR.^18^ In both allo‐ and xenotransplantation, acute AMR is anyway a potentially dramatic event if not properly diagnosed and treated.

According to the current International Society for Heart and Lung Transplantation (ISHLT) working formulation, the diagnosis of cardiac AMR is based on morphological and immune‐pathological abnormalities found on EMBs. The main histopathologic feature of cardiac AMR is microvascular injury, with “activated mononuclear cells” accumulating against interstitial capillary walls, whereas the immunophenotypic characteristics of AMR consist of multifocal C4d staining on ≥50% capillary endothelium or occurrence of ≥10% CD68‐positive cells (macrophages) within capillary walls. Depending on the evidence of only morphological (pAMR1 H+), immune‐pathological (pAMR1 I+), or both (pAMR2) features on an EMB, AMR is regarded as “suspicious” or “definite,” with “severe” AMR (pAMR3) being eventually considered when additional extensive tissue injury is also observed.^19^ In our case, EMB was pAMR2, which we have previously shown to be more likely associated with DSA and overt graft dysfunction than pAMR1.^3^ Nevertheless, the pAMR grading system is only modestly correlated to the outcome, with similar cardiovascular mortality among pAMR1 H+, pAMR1 I+, and pAMR2 patients,^20^ and it does not provide definite guidance for patient management per se.^21^ In fact, the natural history of AMR is more predicted by its clinical phenotype, and treatment recommendations are mainly based on the occurrence of concomitant graft dysfunction whatever underlying pAMR grade.^2^

Actually, the clinical presentation of cardiac AMR ranges from asymptomatic detection on routine EMB to severe allograft dysfunction and cardiogenic shock despite a seemingly similar appearance on EMB.^3^, ^4^, ^5^, ^22^ Overall, the incidence of overt clinical AMR is lower than that of asymptomatic AMR (8.3–47% of cases depending on the definition of AMR and graft dysfunction^2^, ^3^) and intriguingly increases with time from transplantation (9.1% of patients with C4d‐CD68 positive EMBs during the first 4 months vs. 50% during the following 5–12 months,^3^ and 25% during the first year vs. 50% afterward^5^). Furthermore, the outcome of treated symptomatic cardiac AMR is better with early (<1 year) than with late (>1 year) AMR, with 100% 1‐year retransplant‐free survival for early cases versus 65% for late ones.^5^ Hodges et al and Coutance et al found even poorer outcome in their population of late (>1 year) symptomatic AMR, with 50% 1‐year mortality despite therapy.^4^, ^22^ Currently, our comprehension of the pathways regulating graft injury, clinical phenotype, outcome, and response to therapy is too limited to elucidate these observations.^23^ Nevertheless, elements of understanding may come from the field of kidney allotransplantation, where the timing of clinically apparent AMR is also closely related to outcome. Indeed, prompt therapy completely prevented graft loss and enabled graft function to recover to a near‐normal level at 13 months in kidney recipients with early AMR,^9^ whereas estimated glomerular filtration rate (eGFR) progressively and irremediably declined irrespective of the kind of therapy attempted in the large majority of patients with allograft dysfunction and late AMR in another cohort.^24^

As opposed to heart transplantation, early renal AMR is restricted to a very specific phenotype of humoral rejection. Indeed, it occurs within the first 30 postoperative days as an anamnestic response of memory B‐cells in presensitized patients with or without DSA at transplant time. Its clinical course is very aggressive, with an abrupt and intense rise of DSA accompanied by a fast eGFR decline eventually followed by oliguria and graft loss if untreated. By contrast, late AMR usually presents as a more indolent process, with usually no or only mild allograft dysfunction at diagnosis, progressive GFR loss over years, and it is associated with either preexisting or de novo DSA. At the biopsy level, early and late AMR also differ in both morphologic and immune‐pathologic patterns. Whereas early AMR always displays typical features of “active AMR,” late AMR may exhibit both “active” and/or “chronic active” lesions. “Active AMR” is characterized by acute tissue injury (e.g., microvascular inflammation, arthritis, acute thrombotic microangiopathy) and is interpreted as an initial and reversible process in the evolutive course of humoral rejection, while “chronic active AMR,” depicted by transplant glomerulopathy, peritubular capillary basement membrane multilayering or arterial intimal fibrosis, feature more advanced and definitive lesions.^12^, ^25^ Of note, the current ISHLT working formulation does not discriminate between both entities in cardiac AMR^19^ and this gap may account for some of the prognostic variations observed according to time from cardiac transplant.

In addition, early and late kidney AMR also differ in their immune‐phenotypic pattern, with almost invariable C4d deposition in early disease but frequent C4d negative specimen in late cases.^25^ But of importance, the effect of eculizumab in both prevention and treatment of early AMR indicates that the activation of the complement cascade in this particular condition is not only a marker of antibody‐endothelium interaction but also a central pathogenic player in the generation of graft dysfunction and tissue injury. Indeed, regular pre‐emptive infusions of eculizumab during the initial 1–12 post‐transplant months enabled near‐complete AMR avoidance at 1 year in a cohort of 26 recipients of positive crossmatch renal transplants.^8^ Furthermore, Tan et al recently showed that eculizumab (with or without plasmapheresis) administered as a primary therapy in 15 kidney recipients with early AMR prompted a rapid and sustained improvement of allograft function, with the recovery of “active” lesions in 87.3% of cases and no graft loss at 1 year.^9^ In contrast, therapies targeting complement inhibition showed poor efficacy in late AMR, questioning the importance of the complement cascade activation in the generation of tissue injury and graft dysfunction when chronic lesions are established.^12^ Hence, eculizumab was given as a salvage therapy neither reversed the pace of progressive graft dysfunction nor prevented graft loss in five kidney recipients with late AMR,^26^ and eculizumab had at best a mild effect on the eGFR trajectory with no influence on endothelial cell‐associated transcripts in a small unblinded randomized control trial on 10 treated patients and five controls.^27^ Altogether, these findings show how diverse the mechanism of graft injury can be in AMR, with complement cascade activation playing a major role only particularly in early cases.

Notwithstanding post‐transplant timing, our acute AMR case shares many characteristics with early kidney AMR that may hypothetically suggest complement‐dependent tissue injury and sensitivity to therapeutic terminal complement blockade. First, DSA increased extremely abruptly and intensely, with a transition from undetectability to 46,249 MFI in total after 32 days. Although pretransplant screening did not reveal any relevant anti‐HLA antibodies to the implanted organ in our patient, we cannot exclude cryptic pre‐sensitization and late anamnestic response at the time of steroid tapering, especially since pretransplant LVAD use confers a significant risk factor for anti‐HLA antibodies production,^28^ and since de novo DSA usually does not rise at such a pace. Second, the initial rapid decline of allograft function with normal LVEF 32 days earlier appears very close to the clinical phenotype of kidney early AMR. Third, the EMB displayed unambiguous “microvascular injury” lesions that approximate quite well the “active” pattern typical of early kidney AMR with diffuse complement activation and C4d deposition in nearly all capillaries. Fourth, the quick and sustained response to therapy can be seen as a retrospective hallmark of “active” AMR as opposed to “chronic active” AMR. In brief, what our case of late AMR suggests, is that the phenotype of abrupt loss of allograft function conjugated to intense DSA production, microvascular injury, and C4d deposit on EMB may be more reflective of complement‐mediated injury than time from transplant.

To our best knowledge, our case report is the first detailed description of the successful use of eculizumab to treat acute cardiac AMR. It contrasts with the series reported by Law et al, where six of the 11 eculizumab‐treated pediatric heart recipients with AMR expired at a median time of 21 days.^16^ Although many similitudes can be found between their series and our patient (all with DSA, most with hemodynamic compromise), there was the main difference in the timing of eculizumab initiation: a couple of hours from diagnosis in our case versus 0–23 days in Law et al. Indeed, late administration of eculizumab (e.g., as salvage therapy after plasmapheresis or immunoadsorption failed), may occur at a stage where early active lesions already evolved to some form of chronic active AMR, with possible less importance of complement in graft injury mediation and hence less drug benefit.

Finally, the treatments implemented beyond eculizumab and aimed at DSA suppression also likely contributed to the remarkable long‐term outcome of our patient, with no relapsing AMR and no subsequent CAV. Indeed, DSA strikingly decreased over 4 weeks, enabling eculizumab discontinuation after only two doses, and were undetectable 6 months after treatment initiation. The control of DSA production is usually attempted with agents targeting B‐ (e.g., IVIG and Rituximab) and T‐cells (e.g., corticosteroids and thymoglobulin) in different combinations despite few supporting data and conflicting results.^17^ As illustrated here, time from DSA onset to detection may be an important factor determining therapeutic response and early DSA may be easier to suppress.^17^

Prospective multicenter randomized‐controlled studies will be needed in the future to assess the optimal timing of eculizumab administration, to compare its efficacy and safety with more established therapeutic modalities like plasmapheresis, and to precise which clinical profile can best predict treatment benefit. Meanwhile, we believe that our experience added to the recent knowledge on prevention and treatment of AMR with eculizumab may help to reduce the high fatality burden of this syndrome in heart transplantation.

We also believe that experience with eculizumab in allotransplantation may be relevant in discordant xenotransplantation, particularly in the near upcoming trials of pig‐to‐human kidney transplantation. Although the use of organs from triple KO pigs (GalT‐KO, CMAH‐KO, and B4GalNT2‐KO) is anticipated to lessen the risk of hyper‐acute rejection in humans,^29^ delayed active or acute AMR is still possible through de novo production of DSA directed against various antigens like swine leucocytes antigens class I.^1^, ^30^ Furthermore, overexpression of transgenes coding for human complement regulatory proteins like CD46 does not completely prevent complement‐dependent injury in pig hearts exposed to human blood.^31^ Like in allotransplantation, the implementation of strategies combining immediate tissue injury blockade with desensitization will certainly be necessary to overcome AMR and improve xenotransplant survival. In that sense, eculizumab but also other new complement‐blocking or complement‐depleting compounds such as C1 esterase inhibitors, C3b inhibitors, and cobra venom factor will potentially have a role to play in the prevention and/or treatment of xenotransplant AMR^1^.

## References

[1] Meier R , Longchamp A , Mohiuddin M , et al. Recent progress and remaining hurdles toward clinical xenotransplantation. Xenotransplantation. 2021;28:E12681. 33759229 10.1111/xen.12681

[2] Colvin MM , Cook JL , Chang P , et al. Antibody‐mediated rejection in cardiac transplantation: emerging knowledge in diagnosis and management: a scientific statement from the American Heart Association. Circulation. 2015;131:1608‐1639. 25838326 10.1161/CIR.0000000000000093

[3] Yerly P , Rotman S , Nobile A , et al. Time‐dependent specificity of immunopathologic (C4d‐CD68) and histologic criteria of antibody‐mediated rejection for donor‐specific antibodies and allograft dysfunction in heart transplantation. Transplantation. 2015;99(3):586‐593. 24983305 10.1097/TP.0000000000000246

[4] Coutance G , Ouldamar S , Rouvier P , et al. Late antibody‐mediated rejection after heart transplantation: mortality, graft function, and fulminant cardiac allograft vasculopathy. J Heart Lung Transplant. 2015;34:1050‐1057. 25956740 10.1016/j.healun.2015.03.002

[5] Clerkin KJ , Restaino SW , Zorn E , Vasilescu ER , Marboe CC , Mancini DM . The effect of timing and graft dysfunction on survival and cardiac allograft vasculopathy in antibody‐mediated rejection. J Heart Lung Transplant. 2016;35(9):1059‐1066. 27423693 10.1016/j.healun.2016.04.007 PMC5662939

[6] Montgomery RA , Loupy A , Segev DL . Antibody‐mediated rejection: new approaches in prevention and management. Am J Transplant. 2018;18(Suppl 3):3‐17. 10.1111/ajt.14584 29292861

[7] Zhang Q , Hickey M , Drogalis‐Kim D , et al. Understanding the correlation between DSA, complement activation, and antibody‐mediated rejection in heart transplant recipients. Transplantation. 2018;102:e431‐e438. 29916988 10.1097/TP.0000000000002333 PMC6153056

[8] Stegall MD , Diwan T , Raghavaiah S , et al. Terminal complement inhibition decreases antibody‐mediated rejection in sensitized renal transplant recipients. Am J Transplant. 2011;11:2405‐2413. 21942930 10.1111/j.1600-6143.2011.03757.x

[9] Tan EK , Bentall A , Dean PG , Shaheen MF , Stegall MD , Schinstock CA . Use of eculizumab for active antibody‐mediated rejection that occurs early post‐kidney transplantation: a consecutive series of 15 cases. Transplantation. 2019;103:2397‐2404. 30801549 10.1097/TP.0000000000002639 PMC6699919

[10] Schwotzer N , Paganetti G , Barchi M , et al. Upfront use of eculizumab to treat early acute antibody‐mediated rejection after kidney allotransplantation and relevance for xenotransplantation. Xenotransplantation. 2020;27:e12630. 32698246 10.1111/xen.12630

[11] Muller YD , Aubert JD , Vionnet J , et al. Acute antibody‐mediated rejection 1 week after lung transplantation successfully treated with eculizumab, intravenous immunoglobulins, and rituximab. Transplantation. 2018;102:e301‐e303. 29521880 10.1097/TP.0000000000002165

[12] Böhmig GA , Eskandary F , Doberer K , Halloran PF . The therapeutic challenge of late antibody‐meediated rejection. Transpl Int. 2019;32:775‐788. 30955215 10.1111/tri.13436 PMC6850109

[13] Orandi BJ , Zachary AA , Dagher NN , et al. Eculizumab and splenectomy as salvage therapy for severe antibody‐mediated rejection after HLA‐incompatible kidney transplantation. Transplantation. 2014;98:857‐863. 25121475 10.1097/TP.0000000000000298

[14] Patel JK , Coutance G , Loupy A , et al. Complement inhibition for prevention of antibody‐mediated rejection in immunologically high‐risk heart allograft recipients. Am J Transplant. 2021;21:2479‐2488. 33251691 10.1111/ajt.16420

[15] Chih S , Tinckam KJ , Ross HJ . A survey of current practice for antibody‐mediated rejection in heart transplantation. Am J Transplant. 2013;13:1069‐1074. 23414257 10.1111/ajt.12162

[16] Law YM , Nandi D , Molina K , Gambetta K , Daly KP , Das B . Use of the terminal complement inhibitor eculizumab in paediatric heart transplant recipients. Cardiol Young. 2020;30:107‐113. 31875805 10.1017/S1047951119003056

[17] Kobashigawa J , Colvin M , Potena L , et al. The management of antibodies in heart transplantation: an ISHLT consensus document. J Heart Lung Transplant. 2018;37:537‐547. 29452978 10.1016/j.healun.2018.01.1291

[18] Miyagawa S , Yamamoto A , Matsunami K , et al. Complement regulation in the GalT KO era. Xenotransplantation. 2010;17:11‐25. 20149185 10.1111/j.1399-3089.2010.00569.x

[19] Berry GJ , Burke MM , Andersen C , et al. The 2013 International Society for Heart and Lung Transplantation Working Formulation for the standardization of nomenclature in the pathologic diagnosis of antibody‐mediated rejection in heart transplantation. J Heart Lung Transplant. 2013;32:1147‐1162. 24263017 10.1016/j.healun.2013.08.011

[20] Hammond MEH , Revelo MP , Miller DV , et al. ISHLT pathology antibody mediated rejection score correlates with increased risk of cardiovascular mortality: a retrospective validation analysis. J Heart Lung Transplant. 2016;35:320‐325. 26970471 10.1016/j.healun.2015.10.035

[21] Potena L , Moriconi V , Sabatino M , Agostini V , Leone O . Does the antibody mediated rejection grading scale have prognostic prediction? yes, but the picture is still blurry. Curr Opin Organ transplant. 2019;24:265‐270. 31090634 10.1097/MOT.0000000000000652

[22] Hodges AM , Lyster H , McDermott A , et al. Late antibody‐mediated rejection after heart transplantation following the development of de novo donor‐specific human leukocyte antigen antibody. Transplantation. 2012;93:650‐656. 22245878 10.1097/TP.0b013e318244f7b8

[23] Bruneval P , Angelini A , Miller D , et al. The XIIIth Banff conference on allograft pathology: the Banff 2015 heart meeting report: improving antibody‐mediated rejection diasgnostics: strengths, unmet needs and future directions. Am J Transplant. 2017;17:42‐53. 27862968 10.1111/ajt.14112 PMC5363364

[24] Kikić Ž , Kainz A , Kozakowski N , et al. Capillary C4d and kidney allograft outcome in relation to morphologic lesions suggestive of antibody‐mediated rejection. Clin J Am Soc Nephrol. 2015;10:1435‐1443. 26071493 10.2215/CJN.09901014 PMC4527028

[25] Schinstock CA , Mannon RB , Budde K , et al. Recommended treatment for antibody‐mediated rejection after kidney transplantation: the 2019 expert consensus from the transplantation society working group. Transplantation. 2020;104:911‐922. 31895348 10.1097/TP.0000000000003095 PMC7176344

[26] Yelken B , Arpalı E , Görcin S , et al. Eculizumab for treatment of refractory antibody‐mediated rejection in kidney transplant patients: a single‐center experience. Transplant Proc. 2015;47:1754‐1759. 26293046 10.1016/j.transproceed.2015.06.029

[27] Kulkarni S , Kirkiles‐Smith NC , Deng YH , et al. Eculizumab therapy for chronic antibody‐mediated injury in kidney transplant recipients: a pilot randomized controlled trial. Am J Transplant. 2017;17:682‐691. 27501352 10.1111/ajt.14001

[28] McKenna DH Jr , Eastlund T , Segall M , Noreen HJ , Park S . HLA alloimmunization in patients requiring ventricular assist device support. J Heart Lung Transplant. 2002;21:1218‐1224. 12431496 10.1016/s1053-2498(02)00448-5

[29] Martens GR , Reyes LM , Li P , et al. Humoral reactivity of renal transplant‐waitlisted patients to cells from GGTA1/CMAH/B4GalNT2, and SLA class I knockout pigs. Transplantation. 2017;101:e86‐e92. Erratum in: Transplantation. 2018 Feb;102(2):e88. 28114170 10.1097/TP.0000000000001646 PMC7228580

[30] Martens GR , Ladowski JM , Estrada J , et al. HLA class i‐sensitized renal transplant patients have antibody binding to SLA class i epitopes. Transplantation. 2019;103:1620‐1629. 30951017 10.1097/TP.0000000000002739

[31] Abicht JM , Sfriso R , Reichart B , et al. Multiple genetically modified GTKO/hCD46/HLA‐E/hβ2‐mg porcine hearts are protected from complement activation and natural killer cell infiltration during ex vivo perfusion with human blood. Xenotransplantation. 2018;25:e12390. 29536572 10.1111/xen.12390

